# Coformulated Bictegravir/Emtricitabine/Tenofovir Alafenamide for Postexposure Prophylaxis After Sexual Assault: A Retrospective Real-World Study

**DOI:** 10.1093/ofid/ofae436

**Published:** 2024-07-25

**Authors:** Cabrera-López Teresita de Jesús, Pérez-Barragán Edgar, Nava-Campos Carlos Ruben, Toiber-Rodríguez Marla, Rodríguez-Aldama Juan Carlos, Cruz-Flores Raúl Adrián, González-Rodríguez Andrea

**Affiliations:** Department of Gynecology and Care for Victims of Sexual Assault, Clínica Especializada Condesa Iztapalapa, Mexico City, Mexico; Department of Infectious Diseases, Clínica Especializada Condesa Iztapalapa, Mexico City, Mexico; Department of Gynecology and Care for Victims of Sexual Assault, Clínica Especializada Condesa Iztapalapa, Mexico City, Mexico; Department of Mental Health, Clínica Especializada Condesa Iztapalapa, Mexico City, Mexico; Department of Infectious Diseases, Clínica Especializada Condesa Iztapalapa, Mexico City, Mexico; Department of Epidemiology, Clínica Especializada Condesa Iztapalapa, Mexico City, Mexico; Medical Director, Clínica Especializada Condesa, Mexico City, Mexico

**Keywords:** bictegravir/emtricitabine/tenofovir alafenamide, HIV, INSTI, post-exposure prophylaxis, sexual assault

## Abstract

We report the experience of bictegravir/emtricitabine/tenofovir alafenamide for nonoccupational postexposure prophylaxis in sexual assault cases. Between June 2021 and October 2023, 39 individuals completed the 28-day follow-up; 41% experienced some side effects, and 1 person discontinued the drug because of a rash. No individuals seroconverted to HIV during the follow-up period.

In 2005, the U.S. Department of Health and Human Services released its first recommendations for nonoccupational postexposure prophylaxis (nPEP) used to reduce the risk of HIV infection after nonoccupational exposures to blood, genital secretions, and other body fluids that might contain HIV [1]. Since 2014, the World Health Organization (WHO) has recommended that postexposure prophylaxis (PEP) be offered and initiated as early as possible to all individuals with exposures that have the potential for HIV transmission, ideally within 72 hours [2]. No randomized, placebo-controlled clinical trials on nPEP have been conducted for ethical reasons. However, supported by evidence from animal studies on nPEP, as well as with antiretroviral therapy-based prevention interventions, including the prevention of mother-to-child transmission, and observational studies of prophylaxis after occupational and nonoccupational exposures, nPEP based on 3 antiretroviral drugs is currently recommended, including for people who have experienced sexual assault [3, 4]. A regimen consisting of tenofovir and emtricitabine (FTC) or lamivudine, plus a third component, is recommended by WHO or U.S. Department of Health and Human Services guidelines [2, 5]. The integrative strand transfer inhibitors (INSTIs), raltegravir, and dolutegravir, are recommended in these guidelines as a third component of nPEP, but not bictegravir (BIC), a second-generation INSTI. More recently, the AIDS Education and Training Center in the United States has included coformulated bictegravir among its recommendations as an nPEP option [6]. As of 2021, the treatment guidelines in Mexico included the fixed combination of BIC, FTC, and tenofovir alafenamide (TAF) as an nPEP option [7]. However, data supporting this recommendation are still scarce. We report the experience of BIC/FTC/TAF coformulated for nPEP after sexual assault in a real-world setting.

## METHODS

This was an observational, retrospective single-center study of individuals treated at the Clínica Especializada Condesa Iztapalapa, a free healthcare specialized center in HIV prevention and treatment in Mexico City, Mexico. The usual care for people who have experienced sexual assault is in accordance with the national guidelines and is carried out by a multidisciplinary team consisting of general practitioners, gynecologists, infectious disease specialists, nursing, and social workers. Individuals >15 years of age seen between June 2021 and October 2023 who tended to receive nPEP were included. Everyone who presented within <72 hours after the sexual assault and received at least 1 dose of BIC/FTC/TAF was included. The information was obtained through the clinical record. Individuals who completed a minimum of 28 days of follow-up were included in the analysis. Descriptive statistical information was obtained. Continuous variables were described as median (interquartile range). Categorical variables were described as counts and percentages. The study was approved by the Clínica Especializada Condesa Iztapalapa institutional review board.

## RESULTS

Between June 2021 and October 2023, 73 individuals initiated BIC/FTC/TAF following a sexual assault. Thirty-four (46.5%) did not attend subsequent visits, and 39 (53.5%) completed a minimum follow-up of 28 days. The median age was 24 (interquartile range, 18–28). Thirty-three (84.6%) were cisgender women, and 6 (15.4%) were cisgender men; of these, 4 identified as gay men, and all were identified as Hispanic Latino. The main places where sexual assaults occurred were their homes and the streets. All women reported vaginal assault, and 3 also reported anal sexual assault; the men reported anal assault. Seven (17.9%) reported a history of sexual assault in the past (Table 1).

**Table 1.** Characteristics of Individuals Received Postexposure Prophylaxis After Sexual Assault (n = 39)

Age, median (IQR)	24 (18–28)
Female sex, n (%)	33 (84.6)
Substance abuse disorder, n (%)	4 (10.2)
Alcohol consumption, n (%)	22 (56.4)
Previous sexual assault, n (%)	7 (19.9)
Location of the assault, n (%)	
Home	11 (28.2)
Street	9 (23)
Other	19 (48.7)
Type of exposure, n (%)	
Receptive vaginal sex	33 (84.6)
Receptive anal sex	6 (15.3)
Receptive vaginal/anal sex	3 (7.6)
Time from exposure (h), n (%)	
<24	15 (38.4)
25–48	12 (30.7)
49–72	12 (30.7)
Emergency contraception, n (%)	22 (56.4)
Adverse events, n (%)	
Diarrhea/loose stool	3 (7.6)
Dizziness	6 (15.3)
Nausea/vomiting	10 (25.6)
Headache	2 (5.1)
Sleep disorders	3 (7.6)
Rash	1 (2.5)

Abbreviation: IQR, Interquartile range.

Fifteen (38.4%) started PEP within the first 24 hours, and 24 (61.6%) started between 25 and 72 hours later. Antibiotics were prescribed as prophylaxis for sexually transmitted infections in all participants. Sixteen (41%) individuals who received PEP experienced some side effects during treatment, with the most frequently reported being nausea (25.6%), dizziness (15.3%), diarrhea (7.6%), sleep disorders (7.6%), and headache (5.1%). One person reported discontinuing the drug due to a rash during the first week of prophylaxis; her follow-up HIV test was negative. All individuals reported remission of adverse effects at the end of PEP treatment. No individual seroconverted to HIV during follow-up (4–6 weeks from the start of PEP). As routine practice, no laboratory studies during follow-up are performed.

### Patient Consent Statement

The design of this study was approved in accordance with the local standards of Clinicas Especializadas Condesa, which is under the oversight of its medical management and conforms to standards currently applied in the country. All potentially identifying data were encrypted to protect anonymity, and thus informed consent of individual patients was waived.

## DISCUSSION

Although nPEP is currently approved by the WHO and recommended in the main HIV treatment and prevention guidelines, there is still a low level of knowledge and underuse of nPEP, including among people highly vulnerable to acquiring HIV. The implementation of this prevention strategy and its impact has many barriers such as lack of information, limited access, high cost, and adverse effects of the antiretroviral therapy. Individuals who experience sexual assault are a population that should receive nPEP as soon as possible; they should be considered a highly vulnerable population. Similar to other reports, our study mainly includes adult women, which is one of the populations most affected by sexual assaults [5, 8]. Compliance with follow-up was low, but this is not different from other studies where adherence and compliance with follow-up at 28 days can be as low as 38%. This is more common among sexual assault survivors like our study population [8]. The geographic location of the clinic, low educational and economic level, and complex social dynamics could be investigated as possible causes that influenced the loss of follow-up of our population.

Although in Mexico BIC/FTC/TAF is recommended as nPEP, most countries have not adopted this recommendation, mainly because of a lack of data supporting the use of nPEP based on BIC. There are studies with first-generation INSTIs such as raltegravir or elvitegravir, where safety and increased adherence to nPEP have been demonstrated compared to older regimens such as reinforced protease inhibitors, without seroconversions to HIV during follow-up [2, 5, 8]. These studies include individuals who have experienced sexual assault.

More recently, rapid and adequate concentrations of bictegravir in seminal plasma, rectal fluid, and cervicovaginal fluid have been demonstrated, giving it an adequate profile as an nPEP option [9]. In addition, a study in macaques showed that BIC/FTC/TAF induces protection against the acquisition of HIV infection [10]. This has led to clinical studies based on coformulated BIC in humans. One study included 52 individuals, of whom only 3.8% were heterosexual women; there were no HIV infections. The main adverse effects were nausea and diarrhea, and 1 person discontinued therapy [11]. These findings are very similar to those found in our population, highlighting that most of our sample are women. In another open-label, single-arm clinical trial conducted in China, which included 112 participants, there was a high rate of adherence and completion of nPEP; only 1 person discontinued prophylaxis, and there were no cases of HIV infection in the study [12]. Unlike our study, a low percentage of adverse effects is reported here; only 5 people experienced mild effects.

On the other hand, in recent years the use of preexposure prophylaxis (PrEP) has become widespread and new drugs have been approved for PrEP. However, like PrEP, nPEP needs to be disseminated, facilitating access to dispensing centers and a continuum of care focused on people, which leads to improved follow-up rates. Having new therapies with adequate efficacy and safety profiles is also necessary, which includes second-generation INSTIs, and new molecules, such as long-acting drugs, should be considered to guarantee better completion of the recommended days of nPEP.

Our study has some limitations that need to be highlighted. This is a retrospective study with a small sample size and is not the appropriate design to evaluate efficacy and safety. There are limitations in routine follow-up of users, and this led to a large loss of sample to follow-up. However, being a real-world study, the results obtained are the closest to real settings, mainly in low- and middle-income countries. On the other hand, the follow-up in most individuals is up to 28 days, and although the tests performed for the detection of HIV-1 are based on antigen/antibody, longer term follow-up is necessary. Finally, most of the sample are women, and results could vary between men.

There was a large loss to follow-up in this real-world retrospective study of nPEP that primarily included women. Among individuals who completed follow-up, BIC/FTC/TAF had a high rate of PEP completion, no serious adverse events were reported, and there were no cases of HIV-1 seroconversion. Coformulated BIC should be considered as an option for nPEP after sexual assault.

## Notes

***Acknowledgments.*** The authors thank the clinic team that participates in the care of sexual assault victims.

***Patient consent.*** No patient-level data were used in this report.

***Data availability.*** The data sets used and/or analyzed for the current study are available from the authors upon reasonable request.

***Author Contributions.*** Conceptualization: E. P. B. and T. J. C. L.; design of the study: E. P. B. and T. J. C. L.; data acquisition: P. R. B., T. J. C. L., R. A. C. F., and J. C. R. A. E. P. B., T. J. C. L., M. T. R., and J. C. R. A. prepared the article. All authors have read and agreed to the published version of the manuscript.

***Financial support*.** No financial support was received for this study.
